# Phenotype and Genotype Analysis of Chinese Patients with Osteogenesis Imperfecta Type V

**DOI:** 10.1371/journal.pone.0072337

**Published:** 2013-08-20

**Authors:** Zeng Zhang, Mei Li, Jin-Wei He, Wen-Zhen Fu, Chang-Qing Zhang, Zhen-Lin Zhang

**Affiliations:** 1 Department of Orthopedic Surgery, Shanghai Jiao Tong University Affliated the Sixth People’s Hospital, Shanghai, PR China; 2 Metabolic Bone Disease and Genetic Research Unit, Department of Osteoporosis and Bone Diseases, Shanghai Jiao Tong University Affliated the Sixth People’s Hospital, Shanghai, PR China; 3 Key Laboratory of Endocrinology, Department of Endocrinology, Ministry of Health, Peking Union Medical College Hospital, Chinese Academy of Medical Science, Beijing, PR China; Oslo University Hospital, Norway

## Abstract

Osteogenesis imperfecta (OI) type V is an autosomal-dominant disease characterized by calcification of the forearm interosseous membrane, radial head dislocation, a subphyseal metaphyseal radiodense line, and hyperplastic callus formation. The causative mutation, c.-14C>T in the 5'-untranslated region of *IFITM5*, was recently discovered to be involved in this disease. However, in spite of the little genotypic variability, considerable phenotypic variability has been recognized in two cohorts of patients, the majority of whom were Caucasians. Using exome sequencing, we identified the same heterozygous mutation in four Chinese families with OI type V. This study confirms the molecular cause of OI type V and describes the phenotype of Chinese patients with this disorder. In conclusion, the phenotype of Chinese patients was generally similar to that of Caucasian patients.

## Introduction

Osteogenesis imperfecta (OI) is a remarkably heterogeneous disorder characterized by bone fragility and low bone mass, with variably severity ranging from death in the perinatal period to subtle increase in fracture frequency [1]-[4]. Associated features may include blue sclerae, dentinogenesis imperfecta, hearing impairment, progressive deformity of long bones and/or spine, and joint hyperextensibility [5]. According to the latest clinical epidemiology, it is one of the most common skeletal dysplasia groups (0.74–0.79 per 10,000 birth) [6], [7].

Originally in 1979, OI individuals were categorized into four types (type I [MIM 166200], type II [MIM 166210], type III [MIM 259420], and type IV [MIM 166220]) by the Sillence classification system, on the basis of characteristic phenotypes and laboratory findings [5], [8], [9]. The majority of the conditions are caused by heterozygous mutations of either *COL1A1* (MIM 120150) or *COL1A2* (MIM 120160) [10], [11]. However, the heterogeneity of OI is also reflected at the genetic level. A substantial number of individuals with OI do not have a mutation in one of the collagen genes. A growing list of genes that encode proteins involved in the posttranslational processing or modification of type 1 collagen was found to be associated with OI. Mutations in *SERPINF1* (MIM 172860) and *CRTAP* (MIM 605497) were found responsible for OI types VI and VII, respectively [12], [13]. Further mutations responsible for autosomal-recessive OI were identified in *LEPRE1* (MIM 610339), *PPIB* (MIM 123841), *SERPINH1* (MIM 600943), *FKBP10* (MIM 607063), *BMP1* (MIM 112264), and very recently, *WNT1* (MIM 164820) [14]–[20].

In 2000, Glorieux et al. [21] described a novel form of OI with distinguishing clinical and radiological features, which they designated OI type V (MIM 610967). It is dominantly inherited, and is characterized by absence of blue sclera, absence of dentinogenesis imperfecta, propensity to hyperplastic callus (HPC) formation, calcification of the forearm interosseous membrane, radial-head dislocation, and a subphyseal metaphyseal radiodense line. Recently, a recurrent mutation in *IFITM5* (c.-14C>T) has been found to be responsible for OI type V [22], [23]. However, in spite of the little genotypic variability, considerable phenotypic variability has been recognized in two cohorts of patients, the majority of who were Caucasian [24], [25]. Here, we identify the recurrent mutation in *IFITM5* by exome sequencing in a cohort of five Chinese patients with OI type V: two affected individuals from one family and three simplex individuals, and describe the clinical and radiological findings of the disease.

## Materials and Methods

### Ethics Statement

This study was approved by the Ethics Committee of the Shanghai Jiao Tong University Affiliated Sixth People’s Hospital. All the adult participants and the parents of children participants signed informed consent documents before entering the study. All these cases are previously unreported. All patients were diagnosed by their typical clinical and radiographic presentations. *COL1A1* and *COL1A2* were examined by Sanger sequencing and were not found to harbor mutations in these genes.

### Exome Sequencing and Variant Filtering

Exon-enriched DNA was sequenced by the Illumina Genome Analyser II platform following the manufacturer’s instructions (Illumina). Raw image files were processed by the Illumina pipeline (version 1.3.4) for base calling and generating the reads set. The sequencing reads were aligned to the NCBI human reference genome (NCBI36.3) using SOAPaligner [26]–[28]. The SOAPsnp results were filtered as follows: The base quality was equal to or more than 20, and the sequencing depth was between 4 and 200, whereas the estimated copy number was less than two, and the distance between two SNPs was more than 5 bp [29], [30]. Approximately 100 million reads were quantified and mapped to the hs37d5 human reference genome DNA sequence, resulting in an average read depth of 70.0–96.5 for each individual whole exome sequencing. We collected reads that were aligned to the designed target regions for SNP identification and subsequent analysis. The consensus sequence and quality of each allele was calculated by SOAPsnp. The low-quality variations were filtered out using the following criteria: (i) quality score 520 (Q20); (ii) average copy number at the allele site 42; (iii) distance of two adjacent SNPs 55 bp; and (iv) sequencing depth 54 and 4500 [31].

### Sanger sequencing analysis

All exons and their exon-intron boundaries in the *IFITM5* gene were amplified via polymerase chain reaction (PCR). This sequence has been deposited in GenBank with the accession no. CH471278. Direct sequencing was performed using the BigDye Terminator Cycle Sequencing Ready Reaction Kit, v. 3.1 (Applied Biosystems, Foster, CA), and the sequencing was analyzed with an ABI Prism 3130 automated sequencer. SNPs were identified using Polyphred (http://droog.mbt.washington.edu/poly_get.html).

## Results

### Clinical features

**Figure 1:**
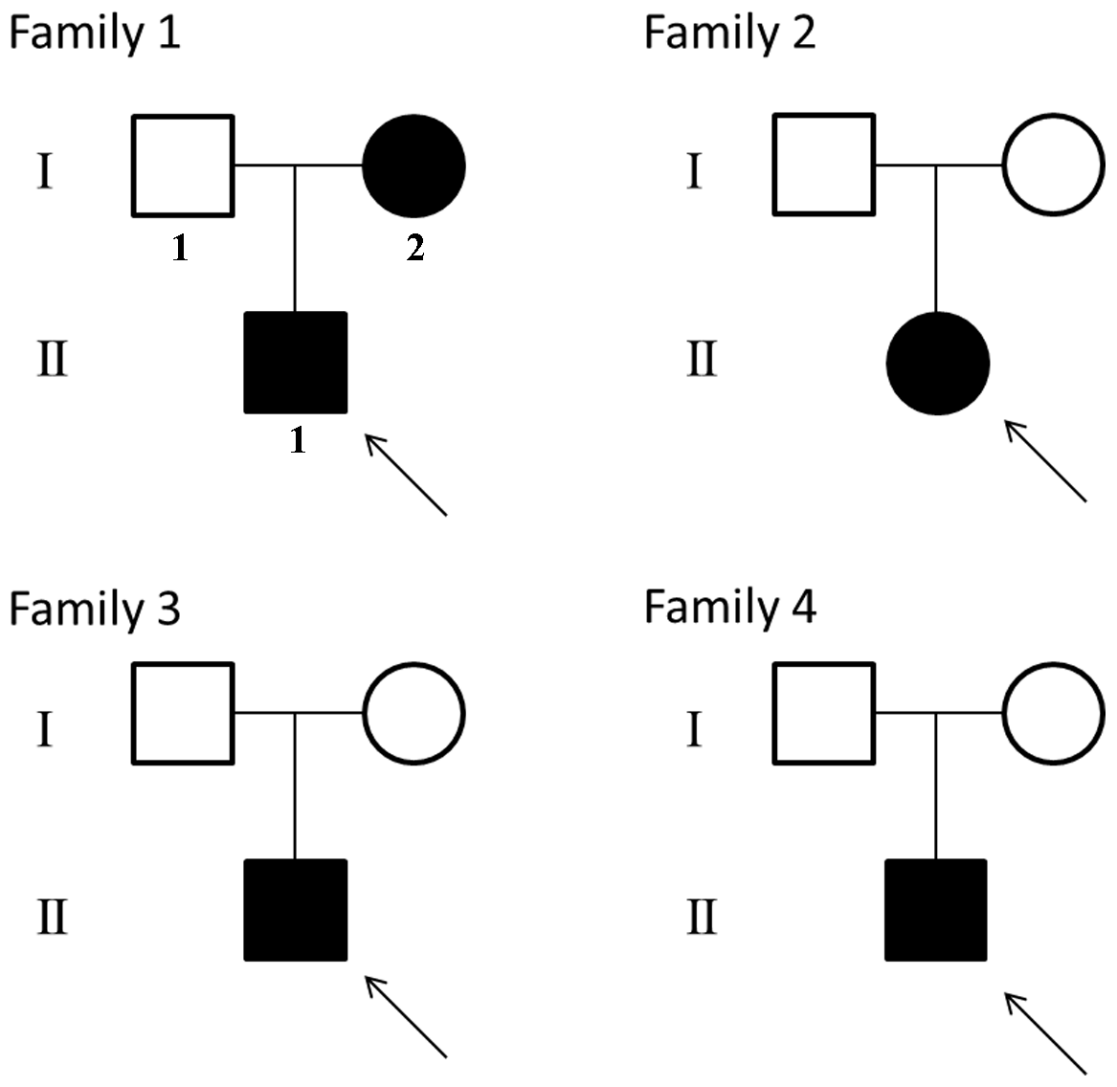
Pedigrees of the four families in this study. Patients with OI type V are shown by filled symbols. Arrows indicate the probands.

All participants were of Han ethnicity. Pedigrees of all of the four families are shown in Figure 1. The major clinical findings are summarized and compared with Caucasian patients [24], [25] in Table 1. Review of the available radiographic documentation revealed that all of the patients had at least one episode of typical hyperplastic callus formation following fracture. Forearm radiographs showed some degree of calcification of the interosseous membrane in patients aged over 14 years but in those aged below 7 years. The interindividual variability in disease severity was quite wide. The wide variability within family 1 was also observed. Detailed history of each family is described as follows.

**Table 1:** Clinical and radiological features of cohort.

Patient	F1- I2	F1- II1	F2	F3	F4	Caucasian patients
Race	Han	Han	Han	Han	Han	
Sex	Female	Male	Female	Male	Male	
Current age, y	29	7	18	4	14	
Positive family history	Yes	Yes	No	No	No	
First fracture age	4 years	3 years	4 years	18 months	14 months	variable
Times of fracture	11	5	6	3	9	variable
Blue sclera	**-**	**-**	**-**	**-**	**-**	**-**
Lumbar spine areal bone mineral density	0.901g/cm^2^	N.A.	N.A.	N.A.	0.571g/cm^2^	Very variable
Dentinogenesis imperfect	**-**	**-**	**-**	**-**	**-**	-
Radial head dislocation	**+**	**-**	**+**	**-**	**+**	Present > 4 years
Calcification of interosseous membranes	**+**	**-**	**+**	**-**	**+**	Present > 4 years
HPC formation	**+**	**+**	**+**	**+**	**+**	Not observed < 9 months
Metaphyseal dense bands	**-**	**+**	**-**	**+**	**-**	Present in young children
Wormian bones	N.A.	N.A.	N.A.	N.A.	**+**	Not described

N.A. represents not available.

### Family 1

**Figure 2:**
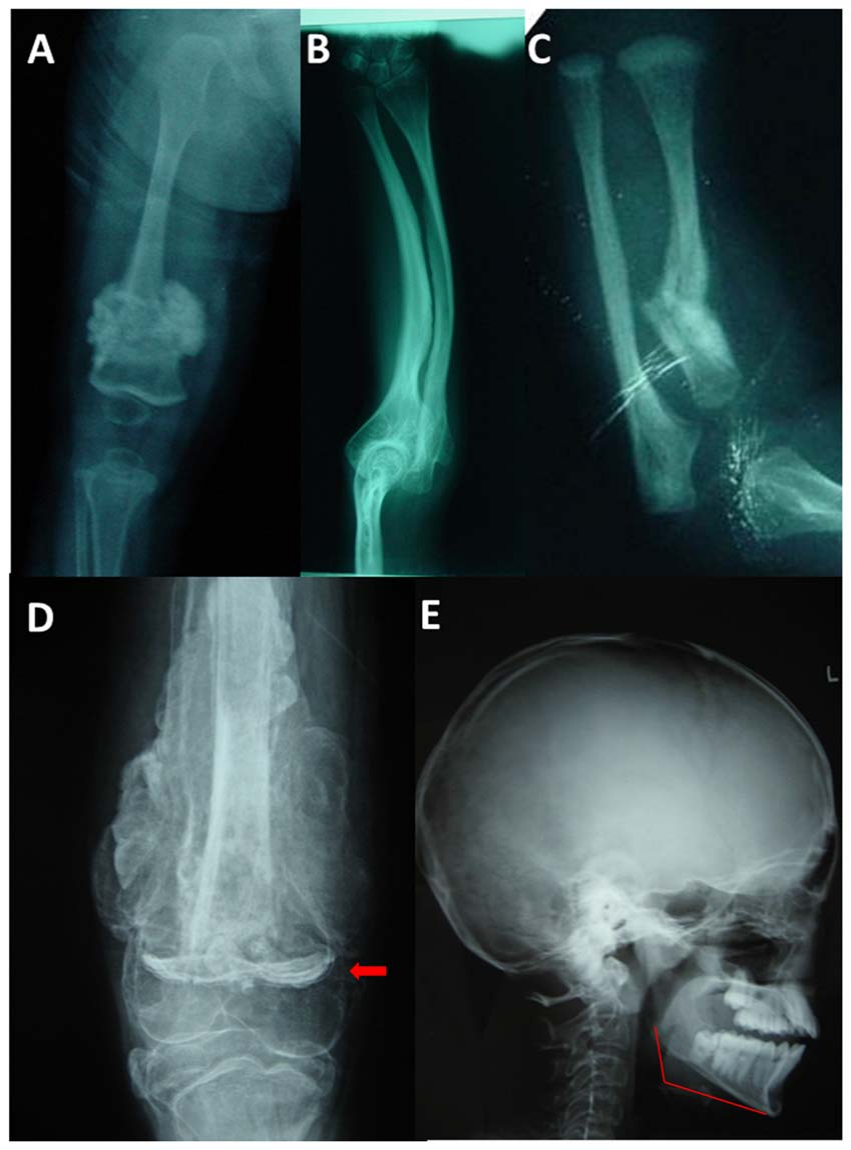
Typical radiographic manifestations. (A) Radiography of the proband of family 1 revealed an irregular radiodense mass arising from the right femur and widened metaphyses with metaphyseal dense bands. (B) Radiography of the affect mother of family 1 revealed calcification of the forearm interosseous membrane and radial-head dislocation. (C) Radiography of the proband of family 3 revealed metaphyseal dense bands of distal radial but no calcification of the forearm interosseous membrane or radial-head dislocation. (D) Radiography of the proband of family 4 revealed an irregular radiodense mass arising from the left femur and “zebra” lines (arrow) induced by intravenous ibandronate. (E) Skull radiography of the proband of family 4 revealed Wormian bones and mandibular hypoplasia (the drawn line represents the normal contour).

The proband (II1) with OI presented to our clinic was a 7-year-old boy. He had experienced his first fracture in the right thigh at the age of 3 years. Radiography revealed an irregular radiodense mass arising from the right femur and widened metaphyses with unusual lucency of the metadiaphyseal regions (Figure 2A). To date, he has had five previous fractures including the thighs and forearms. Birth and fracture histories were obtained directly from his parents. His mother (I2; 29 years of age) also presented with OI, and experienced 11 fractures including the thighs, forearms, and tibiae. Radiography revealed an irregular radiodense mass arising from the right femur, calcification of the forearm interosseous membrane and radial-head dislocation (Figure 2B). Both the proband and his mother did not show blue sclera or brittle teeth, and no hearing loss was evident.

### Family 2

An 18-year-old girl (II1) with OI presented to our clinic with chief complaints of a traumatic progressive swelling and pain in the right thigh. She had experienced her first fracture in the forearm at the age of 4 years. To date, she has had six previous fractures including the thighs, forearms, and arms. Birth and fracture histories were obtained directly from her parents. No family history of bone fragility or consanguineous marriage was identified. She did not show blue sclera or brittle teeth, and no hearing loss was evident. Physical examination revealed local tenderness and swelling in the right thigh. A large bony mass was detected that was fixed to the right femur. Radiography revealed a large irregular radiodense mass arising from the right femur. Bone scintigraphy demonstrated not only an abnormal accumulation in the right thigh but also mild deformities in the shoulders and the right knee.

### Family 3

The proband (II1) was a 4-year-old boy, who was born to a healthy nonconsanguineous couple. The boy had been born at term after an uncomplicated pregnancy. He had experienced his first fracture in the forearm at the age of 18 months. To date, he has had three previous fractures including the left thigh and forearms. Birth and fracture histories were obtained directly from her parents. No family history of bone fragility or consanguineous marriage was identified. He did not show blue sclera or brittle teeth, and no hearing loss was evident. Radiography revealed an irregular radiodense mass arising from the left distal femur and unusual lucency of the metadiaphyseal regions without calcification of the forearm interosseous membrane or radial-head dislocation (Figure 2C).

### Family 4

The proband (II1) was a 14-year-old boy, who was born to a healthy nonconsanguineous couple. The boy had been born at term after an uncomplicated pregnancy. His birth length was 49 cm. He had experienced his first fracture in the forearm at the age of 14 months. To date, he has had nine previous fractures including the left thigh, forearms, and fingers. He received intravenous ibandronate every 3 months for 4 years. Birth and fracture histories were obtained directly from her parents. No family history of bone fragility or consanguineous marriage was identified. He did not show blue sclera or brittle teeth, and no hearing loss was evident. Radiography revealed an irregular radiodense mass arising from the left femur and “zebra” lines induced by ibandronate (Figure 2D). Skull radiography showed Wormian bones and mandibular hypoplasia (Figure 2E; the drawn line represents the normal contour).

### Mutation screening

**Figure 3:**
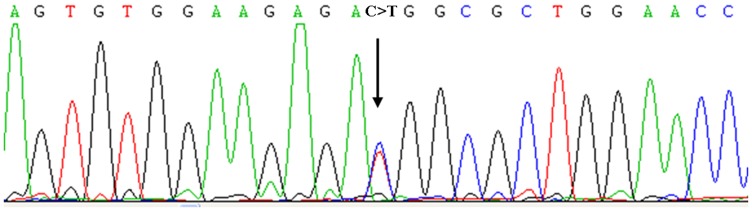
Sanger sequencing of *IFITM5* identified the identical heterozygous c.-14C>T mutation (black arrow) in all the affected patients with IO type V.

We performed exome sequencing on four affected individuals to search for the shared mutations and both unaffected parents of proband 3 to search for de novo mutations. By filtering the data using public SNP databases, we finally identified 28 novel heterozygous variants (i.e., they were not annotated in dbSNP132) which were shared among at least three of four affected individuals and these were analyzed in more detail (Supplementary Table 1). 10 of the 28 variants were de novo variants that was detected in proband 3 but absent in both parents. After further scrutiny of the missense mutations using PolyPhen-2 [32] to predict their likely functional effects, *IFITM5* was the only remaining candidate gene for IO type V. The mutation (c.-14C>T) was predicted to generate an in-frame translation start codon that would add five amino acids (Met-Ala-Leu-Glu-Pro) to the N terminus of IFITM5. By further Sanger sequencing, we identified the same mutation (c.-14C>T) in proband 4 (Figure 3). Furthermore, it was not found in 200 unrelated normal chromosomes from individuals with the same ethnic background. Cosegregation in family 1 and de novo occurrence in the three simplex individuals confirmed that this variation is a disease-causing mutation of OI type V.

## Discussion

The first delineation of OI type V as a distinctive phenotype is attributed to Glorieux et al. in 2000 [21]. Only very recently, genetic researches showed that it is caused by a recurrent heterozygous mutation in *IFITM5* that adds five amino acids (Met-Ala-Leu-Glu-Pro) to the N terminus [22], [23]. The identification of the molecular cause of OI type V provides us an opportunity to reassess the clinical and radiographic phenotype and interpret it with the knowledge of genotype.

Table 1 presents a summary of the clinical findings in the cohort and a comparison with Caucasian patients. The phenotype of OI type V is unique because of the paradoxical combination of an osteoporotic phenotype and exuberant bone formation. The phenotype of Chinese patients was generally similar with that of Caucasian patients, which will be discussed in more detail in the following parts.

For the exuberant bone formation part, the most conspicuous manifestation is HPC formation. In most cases, HPC forms after a fracture, but it may also develop without a history or signs of trauma or fracture. The clinical course of this lesion is self-limited; the lesion regresses slowly and eventually may disappear. The location of HPC has a predilection for femur. Cheung et al. [33] reported that about 50% of HPC occur in femur. All the patients in the present study experienced HPC in their femurs. However, it can also occur in other sites such as humerus. In addition to HPC formation, calcification of the interosseous membrane and periosteal new bone formation are also common manifestations. Calcification of the interosseous membrane of forearm and subsequent radial head dislocation seem to develop gradually and becomes radiologically evident after 4 years of age. Both Rauch et al. [24] and Shapiro et al. [25] reported that all the patients aged 4 years and older exhibited calcification of the interosseous membrane, but patients aged 3 years or younger did not. In our study, patient F1- II1 aged 7 years did not exhibited calcification of the interosseous membrane. Similarly, periosteal new bone formation was also observed only in relatively older patients. These phenotypes generally agree with those of Caucasian patients, but the exact age at which those patients will exhibit calcification of the interosseous membrane may slightly different. Rauch et al. [24] revealed that the formation of new ossified tissue was not always limited to the area of the interosseous membrane, suggesting calcification of the interosseous membrane is not an ectopic calcification but rather represents periosteal new bone formation. Based on these observations, HPC formation, calcification of the interosseous membrane, and periosteal new bone formation seem to be the same pathological process involving the outer surface of bones. HPC formation is more aggressive than the other two manifestations because it often occurs after a fracture when periosteal osteoblast is greatly activated.

For the osteoporotic phenotype part, previous studies have demonstrated that a bone formation defect of trabecular osteoblasts, which may account for the low bone mass and increased bone fragility [21]. A question arises as to whether the specific mutation of *IFITM5* has different effect on trabecular osteoblasts and periosteal osteoblasts.

Another unique feature of OI type V is the presence of metaphyseal dense line. These radiopaque bands are located immediately adjacent to the growth plate, typically in growing children. The metaphyseal dense lines were only observed in patients F1- II1 (aged 7) and F3 (aged 4), which was similar with those of Caucasian patients. It should be noted that there are two other types of metaphyseal dense lines: Harris line assumed to be due to nonspecific events, such as illness and psychogenic stress, and “zebra lines” induced by bisphosphonate therapy as seen in patient F4. The detailed mechanisms of the formation of these lines are still largely unknown. However, the three metaphyseal dense lines are consistent in that they are all formed during periods of rapid growth and may disappear as a result of bone modeling, remodeling, and resorption, implicating that they may share the same mechanism that the function of osteoblast/chondrocyte may overbalance that of osteoclast transiently.

Previous studies found that IFITM5 may play an important role in bone formation, and future studies may elucidate its function. *IFITM5*, also known as *Bone Restricted Ifitm-like protein (BRIL)* [34], is located in chromosomal region 11p15.5 and encodes a 132 amino acid protein, which has two transmembrane domains, such that it has extracellular N and C termini and an intracellular loop [35], [36]. Previous studies showed that the expression of Ifitm5 was restricted to skeletal structures [34], [37], which could explain that patients with OI type V were spared from extraskeletal abnormalities such as dentinogenesis imperfecta or blue sclera. In situ hybridization and immunohistochemistry in mouse embryos showed that the mRNA expression pattern of *Ifitm5* peaked around the early mineralization stage during the osteoblast maturation process and was concordant with bone nodule formation [37]. The previous study has implicated that IFITM5 plays an important role in bone formation, and the future study may elucidate the relationship between the aggressive outward growth nature of HPC formation and the specific 5’ UTR mutation of *IFITM5*. Most probably, the specific mutation enhances the differentiation of osteoblasts, but at the cost of the maturation of osteoblasts. In addition, its expression was detected during embryogenesis [38] as well as in adulthood [34], suggesting Ifitm5 expression is involved not only in bone formation during skeletal development, but also in bone homeostasis. Calcification of the interosseous membrane and periosteal new bone formation may represent gradual outward new bone formation in bone homeostasis. Furthermore, FKBP11 was identified as the only known binding partner of IFITM5, and it was speculated that IFITM5 and FKBP11 might cooperatively regulate bone formation [37]. A better background knowledge of IFITM5 function and the further research on the function of the specific mutation (c.-14C>T) may help us gain a better understanding of clinical picture of OI type V and aid in differential diagnosis and treatment.

In conclusion, the phenotype of Chinese patients was generally similar with that of Caucasian patients. The wide interindividual variability in disease severity was observed even within families. In addition, calcification of the interosseous membrane and periosteal new bone formation develop gradually and become radiologically evident at late childhood.

## Supporting Information

Table S1 **Identified 28 genes containing heterozygous mutations shared among the four affected individuals by exome sequencing.**
(DOC)
