# Supplementary material for: Phenotype and Genotype Analysis of Chinese Patients with Osteogenesis Imperfecta Type V
Source: PLoS One. 2013 Aug 20;8(8):e72337. doi: 10.1371/journal.pone.0072337 (PMC3748067; doi:10.1371/journal.pone.0072337)
Supplement: Table S1 — Identified 28 genes containing heterozygous mutations shared among the four affected individuals by exome sequencing. (DOC) [file pone.0072337.s001.doc]

Table S1. Identified 28 genes containing heterozygous mutations shared among the four affected individuals by exome sequencing

|  | The proband of F1 | I2 of F1 | The proband of F2 | *De novo* mutations of the proband of F2 |
| --- | --- | --- | --- | --- |
| ACSM2A | √ | √ | √ | √ |
| BMS1 | √ | √ | √ | √ |
| FCGBP | √ | √ | √ | √ |
| PKD1L1 | √ | √ | √ | √ |
| AP3S1 | √ | √ | √ | - |
| CCDC66 | √ | √ | √ | - |
| EGFR | √ | √ | √ | - |
| EPB41 | √ | √ | √ | - |
| GNAQ | √ | √ | √ | - |
| GRIP1 | √ | √ | √ | - |
| HERC2P3 | √ | √ | √ | - |
| HSPA5 | √ | √ | - | √ |
| IFITM5 | c.-14C>T | c.-14C>T | c.-14C>T  L29T | c.-14C>T |
| ITIH1 | √ | √ | - | √ |
| KRTAP9-8 | √ | √ | - | √ |
| LYZL2 | √ | √ | √ | - |
| MACC1 | √ | √ | √ | - |
| MYLK | √ | √ | √ | - |
| NCOR1 | √ | √ | √ | - |
| NDUFV2 | √ | √ | √ | - |
| RTKN2 | √ | √ | √ | - |
| RWDD4A | √ | √ | √ | - |
| TRIM48 | √ | √ | √ | - |
| UGDH | √ | √ | √ | - |
| VWF | √ | √ | - | √ |
| WDR72 | √ | √ | - | √ |
| ZNF443 | √ | √ | √ | - |
| ZNF626 | √ | √ | √ | - |
| ZNF676 | √ | √ | √ | - |
